# Community efforts to promote vaccine uptake in a rural setting: a qualitative interview study

**DOI:** 10.1093/heapro/daad088

**Published:** 2023-08-07

**Authors:** Agnes Nanyonjo, David Nelson, Emma Sayers, Priya Lall, Elizabeth Vernon-Wilson, Moses Tetui, Kelly Grindrod, Ros Kane, Mark Gussy, Niro Siriwardena

**Affiliations:** Lincoln International Institute for Rural Health, University of Lincoln, Brayford Pool, Lincoln, Lincolnshire, LN6 7TS, UK; Lincoln International Institute for Rural Health, University of Lincoln, Brayford Pool, Lincoln, Lincolnshire, LN6 7TS, UK; School of Health and Social Care, College of Social Science, University of Lincoln, Brayford Pool, Lincoln, Lincolnshire, LN6 7TS, UK; Lincoln International Institute for Rural Health, University of Lincoln, Brayford Pool, Lincoln, Lincolnshire, LN6 7TS, UK; School of Pharmacy, University of Waterloo, 10 Victoria St S A, Kitchener, Ontario, N2G 1C5, Canada; School of Pharmacy, University of Waterloo, 10 Victoria St S A, Kitchener, Ontario, N2G 1C5, Canada; Department of Epidemiology and Global Health, Umeå University, Umeå, Sweden; School of Public Health Sciences, University of Waterloo, Waterloo, Ontario, Canada; School of Pharmacy, University of Waterloo, 10 Victoria St S A, Kitchener, Ontario, N2G 1C5, Canada; School of Health and Social Care, College of Social Science, University of Lincoln, Brayford Pool, Lincoln, Lincolnshire, LN6 7TS, UK; Lincoln International Institute for Rural Health, University of Lincoln, Brayford Pool, Lincoln, Lincolnshire, LN6 7TS, UK; Community and Health Research Unit, University of Lincoln, Brayford Pool, Lincoln, Lincolnshire, LN6 7TS, UK

**Keywords:** rural health services, vaccine hesitancy, coastal health, qualitative, COVID-19 vaccines

## Abstract

Vaccine hesitancy has been identified as one of the top 10 threats to global health. The causes of low vaccine uptake are many and vary at micro and macro levels. However, rural and remote coastal areas in the UK experience unique vaccine inequalities due to high levels of deprivation and their unique and complex access-related problems. This study aimed to explore community efforts to promote vaccine uptake during the COVID-19 pandemic and understand how the COVID-19 vaccination campaign was experienced by the public. We conducted an exploratory descriptive qualitative study using semi-structured interviews with decision-makers, health professionals and community members in Lincolnshire, a predominantly rural county with a long coastline, a large population of white minority ethnicities, and those living in caravan and temporary housing. Data were analysed using conventional content analysis. Overcoming the various access barriers to vaccination uptake involved working with local media stations, local communities and local community groups, translation of information, bringing vaccines closer to the people through pop-up and mobile clinics and provision of transport and ensuring confidentiality. There is a need to employ inclusive targeted non-conventional care interventions whilst dealing with complex problems as occur in rural and remote coastal regions.

**Contribution to Health Promotion**

- Rural and coastal communities experience socio-economic and health inequalities differently when compared to urban areas.
- Rurality poses challenges in health promotion efforts through multiple access-related barriers.
- This study describes some of the modifications that needed to be made to routine vaccinations programmes to meet the needs of rural and coastal communities.
- The unique challenges faced by rural and coastal populations should be considered in future vaccination and other health promotion campaigns.

## INTRODUCTION

Low vaccine uptake is a pressing global health issue with falling levels of vaccination observed in both adult and children’s programmes (Larson *et al*., 2016). Throughout the course of the COVID-19 pandemic, pockets of low vaccine uptake were observed across the UK, Europe and internationally (Robertson *et al*., 2021; Sallam, 2021; Steinert *et al*., 2022). A majority of those with low vaccine uptake levels in the UK were from poorer households, areas with higher levels of deprivation and areas with larger proportions of ethnic minority populations and medically underserved groups (Robertson *et al*., 2021). While it had been postulated that vaccine refusal rates of 10% and above could hinder the attainment of herd immunity, the thinking has drifted away from herd immunity due to frequent mutations in the SARS-CoV-2 virus (Morens *et al*., 2022). Still, the goal of any vaccination programme is to ensure high levels of uptake (Fine *et al*., 2011; Morens *et al*., 2022).

Historically low vaccination uptake has been attributed to a mixture of individual and health system level factors including poverty, low health literacy, lack of childcare, mistrust of the medical system, one’s value systems, concerns about safety, mis/disinformation and poor access (Fine *et al*., 2011; Mills *et al*., 2020). Vaccination uptake is influenced by both the behaviour of potential vaccine recipients and vaccine communicators highlighting the need to address factors that are beyond the control of individuals while planning vaccination programmes (Williams *et al*., 2020).

From a global perspective, many rural communities experience disadvantage characterized by poor health indicators when compared with their urban counterparts (Scheil-Adlung, 2015; Richman *et al*., 2019). In the UK, rural and remote coastal communities tend to experience high levels of deprivation, health workforce challenges, an ageing population and poor access to health care all of which are intricately connected to the success of vaccination programmes (Bird, 2021; Lloyd and Blakemore, 2021; Nelson *et al*., 2022). The coastal disadvantage was clearly demonstrated in a report by England’s Chief Medical Officer which showed a ‘coastal excess’ of several long-term conditions in comparison to inland towns with similar demographics and deprivation. Tailored interventions are needed to improve the health of coastal populations (Bird, 2021). The experience of health inequalities attributable to rurality tends to be masked by commonly used measures of deprivation. These measures do not take into account distinct rural characteristics, and stressors and the magnitude of rural health inequities can be underestimated. The rural experience of health inequalities ought to be addressed whilst implementing vaccination programmes (Fecht *et al*., 2018; Richman *et al*., 2019).

Access to health care remains central to rural health inequalities yet most measures of inequality tend to measure potential rather than actual access thus underrepresenting access barriers for rural deprived communities. According to behavioural change and health service delivery frameworks, access is not unidimensional but spans availability, accessibility, affordability, adequacy, accommodation and acceptability of health services (Tanahashi, 1978; Obrist *et al*., 2007; Williams *et al*., 2020).

The decision to use a health service (such as whether to get vaccinated) depends on contextual factors and how they interact over time. The interaction between these factors is not always straightforward in rural and remote coastal social spaces which have a unique set of health challenges. At a personal level, individuals need to be empowered to have the capacity to make informed decisions about taking vaccines, given opportunities to take the vaccine in an environment that accommodates their special social norms and circumstances (Michie *et al*., 2011; Williams *et al*., 2020).

Although there is a wealth of knowledge about the factors that affect uptake of COVID-19 vaccines in the UK, studies rarely apply a rural and remote coastal setting lens to their findings. The objective of this study was to explore community efforts to promote the uptake of vaccines in the county of Lincolnshire, a large rural and coastal county in the East Midlands region of England, during the COVID-19 vaccination campaign. The aim of this study was to understand the barriers and facilitators for access to COVID-19 vaccination programmes in a rural and coastal county. It also explored how the COVID-19 vaccination programme was experienced in rural and coastal settings. Specifically, the data collection was guided by the following research questions:

1. What community initiatives were being used to promote the uptake of COVID-19 vaccines in rural and coastal settings?
2. What factors facilitated or prevented access to community-based COVID-19 vaccination programmes in rural and coastal settings?

## METHODS

### Study setting

This study was conducted in the county of Lincolnshire. Lincolnshire is one of the largest rural counties in the East Midlands, UK with both affluent and deprived rural areas as well as hosting a number of coastal communities to the East that are characterized by poor mental and physical health and low levels of health literacy (Bird, 2021). In the UK, rurality is measured using the Rural Urban Classification which is broken down into one of four urban categories (see Table 1) or one of six rural categories (Department for Environment Food & Rural Affairs, 2021). Areas are defined as rural if they have <10 000 inhabitants.

**Table 1:** UK rural urban classifications

Rural classification	Hamlets and Isolated Dwellings
	Hamlets and Isolated Dwellings in a sparse setting
	Village
	Village in a sparse setting
	Town and Fringe
	Town and Fringe in a sparse setting
Urban classification	City and Town
	City and Town in a sparse setting
	Minor Conurbation
	Major Conurbation

### Study design and approach

A descriptive cross-sectional qualitative study was undertaken to explore community efforts to promote vaccine uptake during the COVID-19 pandemic and understand how the COVID-19 vaccination campaign was experienced by rural and coastal communities. We used an empirical–phenomenological approach (Aspers, 2009) placing emphasis on the voice and lived experience of individual stakeholders and health professionals who were working ‘on the ground’ trying to implement and deliver initiatives to improve vaccine uptake during an unprecedented global health crisis. This approach has also been adopted by other qualitative health research during the COVID-19 pandemic (Al Ghafri *et al*., 2020; Collado-Boira *et al*., 2020; Liu *et al*., 2020). Rural and coastal communities can often be neglected or underserved compared with urban counterparts when it comes to health policy and access to services. Furthermore, they have also been shown to have less engagement or opportunities to participate in research (Levit *et al*., 2020). Therefore, this approach was deemed appropriate in exploring a poorly understood aspect of rural and coastal life during a global pandemic.

The findings from the study are reported in line with the Consolidated Criteria for Reporting Qualitative Research (Tong *et al*., 2007).

### Study participants, sampling and recruitment

Participants were included if they were involved in either the management or implementation of the vaccination programme, administering vaccines, mobilizing community members for vaccination, vaccination messaging or if they worked in an area with low vaccine uptake. This included decision-makers at local and national government, vaccination programme implementers (health and social care professionals and vaccinators) as well as community members. The study sample was obtained using a combination of purposive and snowball sampling techniques. Participants were purposively sampled if they belonged to any of the above groups and this was initially done using our existing professional networks with our National Health Service (NHS) and Public Health colleagues where names and email addresses of key stakeholders were provided to the research team via existing professional contacts. Snowball sampling was also employed where we asked interview participants to recommend other suitable colleagues and peers for interview.

### Data collection

Semi-structured online interviews were conducted that ranged from 30 to 60 min. Data were collected between December 2021 and February 2022. All interviews were conducted (by A.N., D.N. and E.S.) online using Microsoft Teams software using a predetermined topic guide (see Table 2) in line with the study aims and research questions. The questions were designed to capture general experiences about efforts to promote COVID-19 vaccine uptake as well as the challenges and successes of delivering these initiatives in rural and coastal areas. Probing techniques (e.g. ‘Could you tell me more about that?’) were used to elicit more in-depth responses based on the participants answers to the initial questions as opposed to pre-existing theory in line with our approach to analysis below (Hsieh and Shannon, 2005). Interviews were digitally recorded with permission and transcribed using the auto transcription function in Microsoft Teams. These were then cleaned and reviewed for accuracy.

**Table 2:** Interview topic guide

Question
1. What are some of the efforts being undertaken in your community to increase COVID-19 vaccine uptake?
2. How do you feel about these efforts?
3. Which of these efforts have been most successful in your opinion and why?
4. Which ones do you feel have not worked so well and why?
5. If you were to improve or change your efforts to build vaccine confidence among more hesitant individuals or groups of people, what changes would you make?
6. Why would make those changes?

### Data analysis

The interview transcripts were imported into NVivo software (NVivo version 12, QSR International Pty Ltd, 2021) to support coding and organization of the descriptive sections of data from each interview. We used an inductive data analysis approach and conventional content analysis to derive meanings directly from the interview data (Hsieh and Shannon, 2005). This approach to analysis is used when the study is aiming to describe a phenomenon, in this instance, community initiatives to improve COVID-19 vaccine uptake in rural and coastal settings. Additionally, another objective of the analysis was to identify barriers and facilitators for vaccine uptake. Transcripts were independently analysed by three members of the research team (A.N., D.N. and E.S.). This begins with reading and re-reading the transcripts to immerse and familiarize themselves with the entire dataset. Following this, the transcripts were then independently coded line by line. Rather than using predetermined categories or codes we allowed these to come from the data. The three researchers then met regularly throughout this process to discuss initial impressions, as well as more detailed interpretation and meaning from the data amongst themselves as well as with members of the wider team. This allowed for consistent opportunities to validate and challenge the individual interpretations of the data. Finally, codes were gathered together in clusters as agreed by the analysts and wider research team. An advantage to this approach is the ability to gain direct information from study participants without the researchers imposing predetermined theoretical perspectives (Hsieh and Shannon, 2005). Study results were also presented to some of the study participants and community members at a dissemination meeting to establish how well they resonated with them.

### Ethical approval

Ethical approval for the study was obtained from the University of Lincoln Research Ethics Committee (Reference UoL2021_7356).

## FINDINGS

Data were obtained from 21 interviews conducted with health care professionals (doctors, vaccinators, midwives, vaccine programme coordinators, care workers, public health authorities and pharmacists: *n* = 15) and community members (community leaders and lay members: *n* = 6) from different work sectors. To ensure improved vaccine uptake, vaccination programme implementers used various approaches that had not been conventionally deployed in delivering routine immunization schedules. Regular vaccination procedures needed to be adapted to accommodate the special circumstances of different population groups living in Lincolnshire.

### Targeted approach, using data to inform which groups to target

As the COVID-19 vaccination programme was being rolled out, it became clear that vaccine uptake was lower in certain populations and geographical areas. A public health official commented that these included communities in areas with high levels of deprivation and areas where a large proportion of the population were not native English speakers and perhaps had limited command of the English language. The majority of this population were from white minority ethnicities of East European origin. Two broad strategies identified by the public health professionals to address these pockets of low vaccine uptake were the need to provide access at a more local level and the need to complement the access with tailored community engagement.

Vaccine hesitancy was reported from social care providers. For example, a care home manager struggled to convince young carers, pregnant carers and carers who were originally from Eastern Europe of the importance of vaccination. He reported that young carers did not perceive themselves as being at risk but with a bit of persuasion, some did ultimately get vaccinated and probably just needed a bit of time. Pregnant carers had concerns about vaccination being the right thing for their babies and once they had delivered their babies, they got vaccinated. He described the difficulties he had convincing some of his employees from Eastern Europe to get vaccinated. According to this respondent, the employees had questioned why they needed to put things in their bodies if they had ‘never had to take a paracetamol in their lives’. He found it necessary to refer them to a doctor who spoke several Eastern European languages for further counselling.

We sent them leaflets that were in Polish, Lithuanian and Latvian and we told them about this doctor. There was a webinar where you could talk to this doctor in [name of town] who could speak Russian, Latvian, Polish, Lithuanian, Portuguese I can’t remember what else, he had one other language. So, to be honest he spoke to them in their own (meaning native) language, or we got other people that have had it done and got them to speak to them. So, we did it by peer group.

Delivering a successful vaccination programme depends on effective public health messaging using the right people and effective communication channels. Different strategies were used throughout the deployment of the vaccination programme.

### Local organizations working with public health and the NHS

Public health professionals partnered with local community groups and businesses to raise awareness in communities about vaccine safety and benefits. A senior Clinical Commissioning Group (CCG) [Clinically led statutory NHS bodies responsible for the planning and commissioning of health care services for their local area] member described how they went to lengths working with local community members, businesses and organizations to distribute leaflets with messages encouraging vaccination together with information about vaccination venues and availability of walk-in sessions.

The market traders last Friday were just amazingly putting the flyers up on their stalls. And yeah, everyone very, very positive about it.

### Using local people or trusted community members

A senior public health professional noted that it was necessary to engage with trusted community members such as community champions—defined as active community members drawing on their local knowledge, skills and experiences for health promotion in their local community. Community champions helped share information and have broader conversations about vaccination reinforcing information that was already being communicated by the national government and local health professionals.

It might be talking to community champions because we know they are in touch with communities and can get the message out, you know far better and more successfully than we could ourselves.

### Translations

There were different Eastern European populations across the county of Lincolnshire who spoke different languages making the translation of key public health into a range of different dialects necessary. An NHS Community Engagement Professional explained how translation had a vital role in community-level efforts to build vaccine confidence.

I would say that working with the translator did really help…the flyers were in four languages, so it’s trying to appeal to as many different language speakers as possible.

While translations played a key role in trying to build vaccine confidence at the community level, they were not without problems as some community members complained of inaccurate translations and lack of empathy in the tone of routine messaging. There were also delays in translation and the provision of updated translated messages as public health professionals and local community groups struggled to cope with the rapidly changing guidelines that were being issued by the government on a regular basis. Community organizations sometimes lacked resources for the translation of materials. Vaccination staff were also concerned that community members were receiving uncoordinated messages from multiple sources. A senior operational staff member told how receiving information from scientists and politicians caused inconsistencies in communication.

The underlying message [about vaccines] didn’t change from the scientists, but government was a bit flipped around a little bit with some of it.

### Using traditional and social media

Varied combinations of media engagement methods were used to convey supportive dialogues around vaccination.

### Traditional media (TV and Radio)

Prior to COVID-19, targeted messages about routine vaccination came from the NHS and were often delivered through letters, flyers and text messaging. However, a public health official described how adjustments needed to be made in public health messaging on vaccination during the COVID-19 pandemic. Some of the supplementary methods or adaptations that were often expressed by health professionals included the use of local radio and television stations.

Obviously, there’s the mainstream media messages, so just you know, starting with kinds of wide communications, via press releases and you know, regular media appearances, so using our mainstream media and linking in with radio like BBC Radio Lincolnshire and Lincs FM.

However, the more conventional methods such as fliers and face-to-face conversations remained the main form of vaccine messaging for pregnant women as was reported by one midwife.

We have posters in the clinics and infographics, so those are used as a visual aid in the clinic, but most of it is done through conversation as we have a couple of vaccines that we recommend during pregnancy, flu and whooping cough. So, we often have the conversation about all three vaccines at most appointments.

### Social media and virtual meetings

At the height of the pandemic, face-to-face meetings were substituted for virtual methods of communication including Zoom meetings, teleconferencing and social media. Health professionals and community members often mentioned that social media such as WhatsApp groups, Facebook groups and Twitter became major influential avenues for passing on vaccine information to target groups.

Yes, certainly so, and I would just point out that we have a communications lead who attends the daily vaccination calls. So, we have had the designated Comms Lead and that might have been messaging through vaccination sites or through social media. We’ve produced flyers as well. More targeted resources that we’ve put out with a commitment to different communities. (NHS CCG engagement lead)

Interview participants noted that social media worked well with younger and digitally literate audiences but not so well with older adults. Social media also played a key role in the collaboration between public health and social and leisure facilities. For example, a public health professional described how increasing numbers of COVID-19 cases amongst young people, led to working with night clubs to communicate public health measures and vaccination:

…Engage with younger people on a day, by day, evening by evening basis. In that sort of way to push the messaging out to them in formats they were familiar with. So, for example, nightclubs we use them. We worked with them so they could put messages on their social media pages about safe clubbing in relation to COVID and rates being higher as they were at that point. And we kind of relied on them a bit because they know their target audience. They have far greater reach than we would ever have. (Public Health Professional)

Despite the usefulness of social media in terms of demonstrating reach and displaying feedback, its impact was partly countered by the spread of mis/disinformation.

Like I said, there is benefit of social media as well, isn’t it? You can get that immediacy of reaction. You can see how many likes you’ve got dislikes you can see immediately the comments that people are firing back at you when you try and get something positive out about vaccinations, so I don’t know. I can’t recall any other specific examples of the types of feedback, but just on reflection I recall that it did kind of add additional evidence to what we thought was the case in terms of, you know, the challenges. (Public Health Professional)

### Increasing physical access to vaccines

The rurality of Lincolnshire meant several physical access barriers were observed by interview participants that needed to be overcome to facilitate vaccination efforts. There were financial challenges associated with travelling long distances to access routine vaccination centres. The organization of services was such that vaccination clinic hours were not convenient for manual job workers. Concerns were raised about the privacy of mobile and pop-up clinics. To address these barriers, a combination of interventions were needed.

### Mobile and pop-up vaccinations

Mobile and pop-up vaccination centres were used to address financial challenges associated with transportation by bringing services closer to the people. An NHS staff member who was redeployed to support the booster programme expressed the feeling of relief brought about by the establishment of these centres on the east coast of Lincolnshire.

There is a constant refrain from people, particularly on the east coast of Lincolnshire that they are being ignored and that everything is happening in the city [Lincoln], ‘nothing ever happens in our community’, so, we need to show communities that there are vaccine clinics near them, sometimes you have to take the services to the people.

A home care manager reported about the challenges they experienced earlier on in the pandemic transporting some of their staff and clients to vaccination centres that were later solved by walk-in clinics.

Well, I think what would have been helpful early on, would be to have more locally readily available vaccinations. You know in the early days we didn’t have the walk-in clinics we didn’t have local ability so distance and getting people to vaccination centres was a barrier. I don’t think that is anymore.

While the mobile and pop-up vaccination centres were considered a solution to the challenges of providing vaccines in the hard-to-reach parts of the county with rural and coastal communities, they had a negative impact in that they escalated ‘vaccination stigmatization’ problems for some factory and farming populations from Eastern Europe. For example, a public health professional commented that amongst factory workers in a local market and port town, some people from Eastern Europe were particularly opposed to vaccination and created peer pressure, abuse and impacts across other groups of factory workers that were more receptive of vaccination.

When we took the vaccine to the factory [there] was some discourse within the diverse groups that work within the factories…So, you think you are going to put an action “let us take vaccines to a factory”. But we did not understand the cultures, values, and beliefs in that little micro group of individuals working at the factories.

### Support with access

A senior public health professional reported that in addition to mobile and pop-up vaccination centres other strategies used to address access problems included financial assistance and provision of transport through the establishment of bus shuttle services transferring people to vaccination centres.

It needs to be more of a query…Is there an issue currently preventing them from getting vaccinated? Is there something we can work together to overcome such as transport or it could be there’s a lot of self-employed people across Lincolnshire that were too worried about taking time off and being ill after the vaccine before Christmas because they were waiting for a lock down. They might be losing a lot of their trade and business but at the same time we are having to be very mindful that it is a personal decision.

Vaccines were administered to people in cars to help with psychological conditions such as a phobia of needles, or anxiety about public settings and crowds. A health care manager reported that while this was successful with increasing vaccine uptake; it was also very resource-intensive compared with other vaccination sites.

We have struggled with people with mental health issues, sometimes we’ve got people to the site five or six times. If they are needle phobic, they get as far as the site and then. They don’t want to have it, so we’ve had to communicate quite a bit with them to let them know we can do some in their cars so that they don’t have to come out.

### Using different methods at different times as well as consistency with what worked in the past

One key aspect of the approach used to address barriers to access was the coordinated and willingness to adapt responses to community needs using multiple methods. Programme implementers deployed different approaches according to the messages that needed to be communicated as well as the target group. For example, older adults were more likely to be targeted using printed materials or traditional forms of media while younger people were approached via social media. The choice of communication method was informed by what worked well in the past to reach out and engage with local communities. Examples of tried and tested methods included using social media groups and working with local businesses or community leaders who had established and trusted relationships with the county council and NHS prior to the pandemic.

Crucially, it was suggested the message had to be kept as simple as possible and ‘straight to the point’ when communicating with people who had poor literacy and reading skills. This was mentioned as being a salient concern when working with coastal communities. It was reported that some non-native English speakers had poor levels of literacy in their first language and so assumptions that their native language posed no problem needed challenging. This difficulty was illustrated in a comment by a health professional working on health equalities:

One of the things we did highlight was about illiteracy. Amongst some people coming to England who were illiterate in their own country, never mind them having to speak English.

## DISCUSSION

The findings from this study highlight the complexity of delivering care in Lincolnshire a county that is typical of a rural and remote coastal setting. Rural and remote coastal areas are characterized by diversity of population and geography and limited financial and physical access, which, combined, make implementation of health interventions challenging when compared with urban areas (Local Government Association & Public Health England, 2017; Bird, 2021). The challenges that were experienced by health professionals during the deployment of the COVID-19 vaccination programme are not unique to vaccination programmes but reflect those problems experienced when implementing other health programs in similar settings. Thus, the findings from this study reinforce the role of complexity science in research and the role of hyper-local effects in implementation of programmes in rural and remote coastal settings.

According to complexity science, health systems are complex adaptive systems made up of many self-organizing components that are capable of responding to their own environments and that of others (Plsek and Wilson, 2001; Paina and Peters, 2012). The system is a network of relationships and interactions that occur between individual components of the system to form a functional unit. Interactions occurring within the system as a whole are perceived to be more important than discrete actions and functions of the individual components (Plsek and Wilson, 2001; Paina and Peters, 2012). However, the complexity of health systems is exacerbated by rurality, which not only poses complex challenges but also worsens the experience of many rural residents while using the health system (Harvey and Jones, 2022).

The complex nature of the factors that affect rural and remote settings dissuades implementers from addressing challenges in isolation as evidence suggests such approaches yield little to no effects for the system as a whole and may at times have unintended consequences (Van Beurden *et al*., 2013; Harvey and Jones, 2022). Our findings demonstrated that while bringing services closer to the people through pop-up clinics worked well for coastal communities, it had unintended consequences among factory workers. Different adjustments needed to be made to the delivery approaches to create the same improvement. In the UK, there is limited research on the rural experience of living in diverse remote farming areas, small market towns and coastal villages (Local Government Association & Public Health England, 2017). More research framed within complexity science is needed to generate evidence on interventions that work in the local context for deployment of vaccination and other health programmes. There is also a need to implement more hyperlocal interventions oriented around community concerns and solutions.

Increasing access to vaccination in rural settings is known to be multi-dimensional in nature requiring increased availability, physical accessibility, affordability, adequacy, accommodation and acceptability of health services (Obrist *et al*., 2007). Improving access goes beyond the availability of vaccines to counter other barriers to access such as transport difficulties, and limitations attributable to lifestyle, culture and work patterns (Local Government Association & Public Health England, 2017). Our findings showed that increasing vaccination access through mobile clinics and pop-up centres was not sufficient on its own to increase vaccine uptake. Instead, it was necessary for public health professionals to use evidence-based decision-making by looking at both local data and engaging with local communities to identify pockets with unmet needs and develop culturally adopted interventions.

Careful, granular inspection of local data need contextualization with health professionals’ knowledge of communities along with a willingness to try and understand the underlying social determinants of vaccine hesitancy and committed attempts to address them.

Throughout the pandemic, social media became an important avenue for dissemination and consumption of information (Tsao *et al*., 2021). However, the benefits of employing social media for public health messaging are partially offset by promoting information sharing through poorly regulated channels. Mis/disinformation, which was at times propagated and amplified through social media networks, was a major cause of vaccine hesitancy requiring multicomponent and dialogue-based interventions tailored by target population and context (Loomba *et al*., 2021; Peters, 2022). Our findings are in concordance with the acknowledged important role of social media. Our data show it was effectively used to target population groups through WhatsApp and Facebook groups as well as through official Facebook pages of public health organizations. The findings also concur with other known advantages of using social media such as monitoring reach and getting feedback through scanning social media comments (Goel and Gupta, 2020). Thus, while social media is useful for health communication, a balance must be found as, there is mounting evidence that social media networks have been used for propagating negative propaganda about vaccinations and other health programmes. This balance should be consideration by vaccination programme implementers while designing social media-based interventions (Wang *et al*., 2019; Muric *et al*., 2021; Suarez-Lledo and Alvarez-Galvez, 2021). Although there is limited research on how misinformation propagated on social media networks can be addressed, tentative findings show that using simple tools such as fact checking and debunking misinformation, strategies used by health professionals have at times had positive effects on attitudes towards vaccination (Zhang *et al*., 2021). This reinforces the need to engage with the public they serve by striving for high visibility and credibility on public health agencies on social media.

The strengths of this study included the collection of views across a broad range of participants spanning different occupations, neighbourhoods and ethnic groups, and presentation of the results to community members to ascertain how well they resonated with them. The study approach ensured that the results are trustworthy and are transferable to other settings. Study limitations included difficulty in recruiting vaccine-hesitant individuals and minority ethnicities who were also hesitant to participate in research. Nevertheless, some of the study participants were community leaders conversant with views that are widely prevalent in their communities, including those with varying degrees of confidence in the safety and efficacy of COVID-19 vaccines.

### Methodological reflections

Study strengths included the collection a broad range of views from various stakeholders and presentation of the results to the stakeholders to ascertain how well they resonated with them. Key limitations included challenges in recruiting vaccine-hesitant individuals and difficulties with engaging with minority groups who often preferred to be represented by their trusted community leaders. Nonetheless, the study provided insights into approaches to engaging with rural and coastal communities.

## CONCLUSION

Rural populations experience challenges in access to vaccination and other health programmes differently when compared to the rest of the population. Their unique setting needs to be considered when designing health promotion interventions. Specifically, inclusive targeted non-conventional care interventions are needed to deal with complex problems that occur in rural and remote coastal regions.
